# Effect of aerobic exercise and low-carbohydrate high-fat diet on glucose tolerance and android/gynoid fat in overweight/obese women: A randomized controlled trial

**DOI:** 10.3389/fphys.2023.1056296

**Published:** 2023-01-24

**Authors:** Thorhildur Ditta Valsdottir, Bente Øvrebø, Thea Martine Kornfeldt, Sigbjørn Litleskare, Egil Ivar Johansen, Christine Henriksen, Jørgen Jensen

**Affiliations:** ^1^ Institute of Physical Performance, Norwegian School of Sport Sciences, Oslo, Norway; ^2^ Department of Health Sciences, Kristiania University College, Oslo, Norway; ^3^ Department of Sport Science and Physical Education, University of Agder, Kristiansand, Norway; ^4^ Department of Nutrition, Exercise and Sports, University of Copenhagen, Frederiksberg, Denmark; ^5^ Department of Sports and Physical Education, Inland Norway University of Applied Sciences, Elverum, Norway; ^6^ Institute of Basic Medical Sciences, Department of Nutrition, Faculty of Medicine University of Oslo, Oslo, Norway

**Keywords:** low-carbohydrate high-fat diet, exercise, cardiorespiratory fitness, glucose tolerance, insulin resistance, android/gynoid fat, HOMA-IR, Matsuda ISI

## Abstract

The study was designed to compare the effects of weight loss induced by a low-carbohydrate-high-fat diet or a normal diet, with and without exercise, on glucose tolerance measured as area under the curve (AUC), and android (A) and gynoid (G) fat distribution. The study was registered at clinicaltrials.gov; NCT04100356. In total, 57 women classified as overweight or obese (age 40 ± 3.5 years, body mass index 31.1 ± 2.6 kg/m^2^) were randomly assigned and completed a 10-week intervention using a low-carbohydrate high-fat diet or a normal diet, with or without aerobic interval exercise. An equal deficit of 700 kcal/day was prescribed, either restricting the diet only, or moderately restricting diet and including three 50-min high-intensity bicycle sessions per week. There were thus four groups: normal diet (NORM); low-carbohydrate-high-fat diet (LCHF); normal diet with exercise (NORM-EX); and low-carbohydrate-high-fat diet with exercise (LCHF-EX). Linear mixed models was used to assess differences between groups. With all groups pooled, the intervention resulted in a weight loss of 6.7 ± 2.5% (*p* < 0.001). The intervention did not result in differences between groups in AUC glucose, nor in fasting glucose or indicis for insulin resistance such as Homeostatic Model Assessment, Matsuda Insulin Sensitivity Index, insulinogenic index and disposition index. Post-intervention android fat was lower in LCHF than NORM (3,223 ± 727 vs. 2,533 ± 535 g, *p* = 0.041). LCHF reached a lower A/G ratio than NORM (0.94 ± 0.12 vs. 1.04 ± 0.09, *p* = 0.011) and LCHF-EX (0.94 ± 0.12 vs. 1.09 ± 0.09, *p* < 0.001) after the intervention. LCHF resulted in lower android fat mass compared to NORM and the lowest A/G ratio compared to the other matched groups, but with no accompanying improvement in AUC glucose. In conclusion, although all groups achieved improvements in glucose tolerance, no superior effect was observed with the LCHF diet, neither with nor without exercise.

## 1 Introduction

Overweight and obesity are major risk factors for cardiometabolic disorder, which is associated with cardiovascular disease (CVD) and type 2 diabetes mellitus (T2DM) (Kusminski et al., 2016; Lüscher, 2019). Impaired glucose tolerance and impaired fasting glucose, also termed pre-diabetes, often go undiagnosed and put patients at increased risk of developing T2DM within a few years. The global prevalence of diabetes in 2021 was estimated to be 10.5% and is expected to rise to 12.2% in 2045 (Sun H. et al., 2022).

While the key risk factors for impaired glucose tolerance are being overweight/obese and lack of physical exercise, fat distribution seems to be important, as android central abdominal adiposity shows the strongest correlation with markers of insulin resistance and reduced glucose tolerance (Yusuf et al., 2005). In contrast, gynoid peripheral gluteal and femoral adiposity is associated with better insulin sensitivity and glucose tolerance (Wiklund et al., 2008; Lumish et al., 2020). Calorie deficit resulting in weight loss has a positive effect on glucose tolerance (Norris et al., 2005; Schenk et al., 2009), and intensive lifestyle interventions with focus on behavior, nutrition and activity, have shown superior weight loss and larger improvements in insulin sensitivity in patients with both T2DM and who are overweight/obese, compared with Diabetes Education and Support (self-management) (Pi-Sunyer, 2014). Lifestyle interventions for overweight and obese subjects that result in a mean weight loss of ∼7% can reduce the risk for T2DM, with a 16% reduction in diabetes risk per kilogram of weight loss (Hamman et al., 2006). Moreover, lifestyle interventions for individuals with pre-diabetes have been shown to cease the progression to T2DM, and the positive effect of lifestyle changes and weight loss on insulin resistance has been recognized. In studies by Perreault et al. (2012) and Dagogo-Jack et al. (2022), nearly half of the subjects reverted to normal glucose tolerance, and glycemic decrease was observed after 6 months.

Weight loss is effective in preventing pre-diabetes and T2DM, and the positive effect of exercise bouts on glucose tolerance is well known (Jenkins and Hagberg, 2011; Bird and Hawley, 2016; Malin et al., 2016; Weiss et al., 2016; De Strijcker et al., 2018; Jelstad et al., 2019). However, the improvement in glucose tolerance after exercise bouts is transient and lasts for ∼72 h (Bird and Hawley, 2016). Exercise without weight loss seems to have a smaller effect on glucose tolerance in participants who are overweight/obese, as the baseline glycemic control tends to be poorer in this population (Bird and Hawley, 2016). Methods for extended improvements in glycemic control are of great interest to cease the progression of pre-diabetes to T2DM, and the combination and timing of macronutrients has gained increasing attention in recent years (Hutchison et al., 2017; Nesti et al., 2019; Aoyama and Shibata, 2020; Kolnes et al., 2021). Since the 1970s, low-carbohydrate high-fat (LCHF) diets have been popular to achieve weight loss and improve metabolic health, including glucose tolerance in individuals with overweight and obesity (Noakes and Windt, 2017; Seid and Rosenbaum, 2019). LCHF diets, especially ketogenic diets, have been successful in improving glycemic control (Emadian et al., 2015; Hall and Chung, 2018), lowering fasting insulin and glucose levels (Hall and Chung, 2018; Michalczyk et al., 2020), and reducing glucose surges after a glucose load (Krebs et al., 2013; Hall and Chung, 2018). However, it remains unclear whether a weight loss achieved with the combination of aerobic endurance exercise and an LCHF diet may have an additive effect and result in even larger improvements in glucose tolerance. Therefore, the primary aim of this study was to explore the effect of an LCHF diet and aerobic endurance exercise on glucose tolerance. A secondary aim was to determine whether a certain combination of diet and aerobic endurance exercise affected the distribution and amount of android and gynoid fat.

## 2 Methods

### 2.1 Design and setting

We conducted a 10-week, randomized, parallel group, controlled trial with a 2 × 2 factorial design, where the effect of diet in combination with exercise was studied. The 2 × 2 factorial design generated intervention groups in terms of absence (−) or presence (+) of LCHF diet and exercise. Participants were randomly allocated to one of the four following groups: normal diet only (−/−; NORM), LCHF diet only (+/−; LCHF); normal diet and exercise (−/+; NORM-EX), and LCHF diet and exercise (+/+; LCHF-EX). All participants provided written informed consent. The study was approved by the Regional Committee for Medical Research Ethics in Norway (2013/1529) and conducted according to the Declaration of Helsinki. The study was registered at www.clinicaltrials.gov as NCT04100356. The intervention was conducted at Atlantis Medical College and the Norwegian School of Sport Sciences in Oslo, Norway (January to April 2014). The results in this manuscript are a part of a larger project. A detailed description of the methodologies and other data has previously been published elsewhere (Valsdottir et al., 2020). Some of the previously presented data is included to facilitate the interpretation of current results and strengthen the contextual relevance of the study.

### 2.2 Participants

A total of 199 individuals volunteered to participate in the study and were screened for eligibility. Inclusion criteria were sedentary premenopausal Caucasian women, aged 33–47 years (Bacon, 2017; Patel and Dhillo, 2022; Talaulikar, 2022), body mass index (BMI) 26.5–36.5 kg m^−2^, living close to or in Oslo. Exclusion criteria were pregnancy or breast-feeding, previous medical history of CVD, diabetes, endocrine disorder, kidney disease, smoking or tobacco use, and use of lipid-lowering or diabetes medication. After the initial screening, 60 eligible participants were included in the study.

### 2.3 Randomization

The research leader and assistant performed a computer-generated randomization after baseline measurements (www.randomizer.org). Group allocation was e-mailed to participants immediately after randomization and neither researchers nor participants were blinded.

### 2.4 Intervention

All intervention groups received a calorie deficit prescription of 4,900 kcal week^−1^, achieved by reducing intake in the diet-only groups (NORM and LCHF), and by reducing intake and performing exercise in the diet-exercise groups (NORM-EX and LCHF-EX). The total energy expenditure (TEE) was estimated using the Harris-Benedict equation (Flack et al., 2016) multiplied by a physical activity lever (PAL) coefficient (Ategbo et al., 2005).

### 2.5 Exercise sessions

The exercise groups attended indoor bicycle sessions three times a week, where the main goal was an energy expenditure of 500 kcal. The exercise program was 7 × 4-min intervals at 82%–90% of peak heart rate (HR)with a 3-min active recovery period (∼60% HR_peak_). Polar heart rate monitors (RCX, Polar Electro Oy, Kempele, Finland) were used to record HR and estimate energy expenditure during sessions. The heart rate monitors estimate energy expenditure through algorithms made by Polar^®^. The Polar algorithms are based on previous studies (Byrne et al., 2005), and include measurements of heart rate, and individual information such as weight, height, age, gender, resting heart rate, maximum heart rate, and maximal oxygen uptake.

### 2.6 Dietary counseling

All participants were provided with individual dietary targets and supervised by nutritionists. The nutritionists assessed the food registration, and advice was provided for food and beverages according to the respective group. Each participant had an individual follow-up twice a week *via* Skype, phone, or e-mail. A standard operating procedure was used to secure similar guidance for all participants. The intervention groups had a closed group on social media where they could share information, troubleshoot common nutritional issues, and increase motivation and compliance. Bodyweight was measured every 2 weeks and calorie goal was adjusted to obtain required weight reduction and adherence to diet.

In addition, adherence to LCHF diet was monitored by measuring and reporting ketone bodies. Ketone bodies were estimated in morning urine (Ketostix 2,880, Bayer, Berlin, Germany) as ketosis indicates diet compliance. The ketone scale defined by the manufacturer is as follows: Trace (0.5 mmol L^−1^), small (1.5 mmol L^−1^), moderate (4 mmol L^−1^) and large (8–16 mmol L^−1^).

### 2.7 Diets

The composition of macronutrients in the normal diet groups was according to the recommendations outlined by the Norwegian Health Authorities (Norwegian Directorate of Health, 2014), emphasizing composition with 10%–20% of energy (E%) from protein, 25–40 E% from fat and 45–60 E% from carbohydrates. The composition of macronutrients in the LCHF groups consisted of very low proportions of carbohydrates and high proportions of fat. The first week allowed carbohydrate consumption of 20 g day^−1^ (Atkins, 2002; Foster et al., 2010; Saslow et al., 2014), which equals ∼5 E% of carbohydrates. During the following weeks, the carbohydrate intake was increased by 10 g week^−1^ until the participants reached a maximum of 100 g day^−1^ (Atkins, 2002; Foster et al., 2010). Fat intake was prescribed to ∼70 E% at the beginning of the trial. A proportional increase in carbohydrate intake, alongside a decreased fat intake, was planned throughout the first 9 weeks of the intervention. The protein intake was targeted at 25 E% throughout the trial.

### 2.8 Study procedures

Baseline data collection started 3 weeks prior to the intervention. All tests and measurements were repeated after the 10-week intervention. Throughout the intervention, weight was measured every second week to assess weight loss, using a Bioelectrical Impedance Analysis device (BIA, MC 180 MA Multi Frequency, Tanita, Tokyo, Japan). During the 10-week intervention all food and beverage were weighed on a digital scale, and dietary records were kept every day in online food diary and controlled by nutritionists and adjustments and suggestions were performed when needed.

### 2.9 Outcome measurements

#### 2.9.1 Anthropometric measurements

A wall-mounted stadiometer (Seca 206 Stadiometer Wall Mounted, Seca, Deutschland, Hamburg, Germany) was used to measure height to the nearest 0.5 cm. Weight was measured using a BIA device (MC 180 MA Multi Frequency, Tanita, Tokyo, Japan).

#### 2.9.2 Android and gynoid fat

**FIGURE 1:**
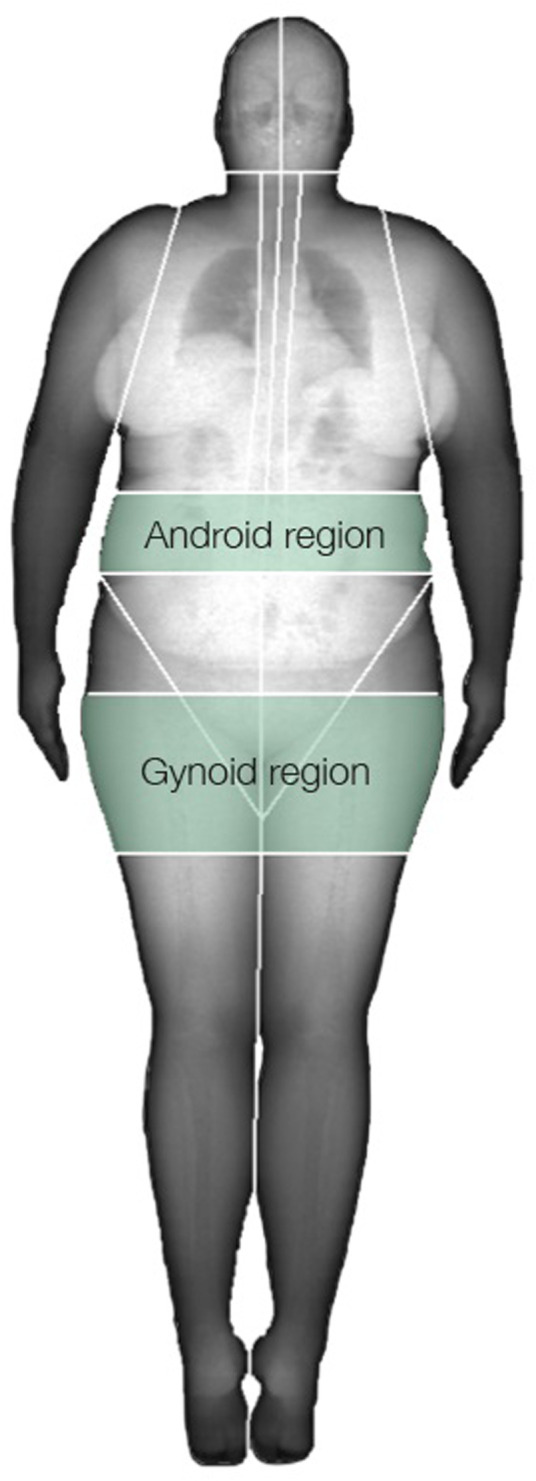
Android and gynoid regions.

A DXA scan was performed in the morning in the fasted state to analyze android and gynoid fat (Lunar iDXA, GE Healthcare, Madison WI, United States). The android area was defined as the area between the ribs and the pelvis enclosed by the trunk region (Stults-Kolehmainen et al., 2013). The upper line is ∼20% of the distance between the iliac crest and the neck. The lower line is the top of the pelvis enclosed by the trunk region. The gynoid region includes the hips and the upper part of the thighs, where both leg and trunk regions are overlapped. The upper line of the gynoid region is below the top of the iliac crest at a distance of ×1.5 the android height, and the total height of the gynoid region is ×2 the height of the android region (Figure 1).

#### 2.9.3 V̇O_2peak_ and HR_peak_

Testing of peak oxygen uptake (V̇O_2peak_) was performed using an incremental test on an ergometer bicycle (Excalibur Sport Cycle Ergometer, Lode, Netherlands). An automatic O_2_/CO_2_ analyzer (Moxus Modular Metabolic System, AEI Technologies, Inc.) was used to measure oxygen consumption and carbon dioxide production. The test started at 50 W and increased by 15 W every 30 s until exhaustion. Determination of V̇O_2peak_ was based on the following criteria: 1) an increase of <1 mL kg min^−1^ after two increments in workload, 2) respiratory exchange ratio (RER) > 1.10, and 3) blood lactate (BLa) > 7 mmol L^−1^. Heart rate was continuously recorded with a heart rate monitor (RCX3, Polar Electro Oy, Finland) during the V̇O_2peak_ test and the highest heart rate (HR_peak_) was noted for each participant.

#### 2.9.4 Fasting glucose and oral glucose tolerance test

Participants arrived at the laboratory at 06:00 after a 12-h fast, and 36 h after the last exercise session. An intravenous catheter was inserted in the antecubital vein, and fasting glucose and insulin samples were collected. After fasting samples (0 min), participants ingested 75 g of glucose dissolved in 300 mL water, within a 5-min time frame. During the oral glucose tolerance test (OGTT), glucose and insulin samples were collected after 20, 40, 60, 90, and 120 min. Blood samples were collected in serum separator tubes (Vacutainer SST 8.5 mL, BD, Franklin Lakes, NJ, United States) and coagulated for 30 min at room temperature before centrifugation (2500rpm at 4°C for 10 min, Eppendorf 5072R, Hamburg, Germany). Samples were stored at 4°C for 3 h before analysis at Fürst Laboratory, Oslo, Norway (Advia Centaur XPT, Siemens Medical Solutions Diagnostics, Tokyo, Japan). Area Under Curve (AUC) for glucose and insulin were calculated with the trapezoid method.

#### 2.9.5 Insulin resistance indicis

Fasting samples and OGTT were used to calculate HOMA-IR, and OGTT was used to calculate Matsuda ISI, the insulinogenic index, and the disposition index. Insulin resistance cut off-values for HOMA-IR and Matsuda ISI were set to 2.29 and 5, respectively (Radikova et al., 2006), whereas the cut-off for the insulinogenic index was set to ≥0.4 (Aono et al., 2018).

### 2.10 Study outcomes

The primary outcome of the study was glucose tolerance measured as area under the curve (AUC), registered in clinicaltrials.org (NCT04100356). Secondary outcomes presented here are android and gynoid fat distribution. Secondary outcomes previously published are body composition, CVD risk factors and cardiorespiratory fitness (Valsdottir et al., 2020).

### 2.11 Sample size calculation and statistical analysis

Sample size was calculated using an online calculator (http://www.math.yorku.ca/SCS/Online/power/). The sample size needed was based on glucose tolerance measured as AUC, using results from previous studies in our lab on exercise and metabolic improvements (Jelstad et al., 2019; Valsdottir et al., 2019), as well as other results on metabolic improvements during weight loss (Snel et al., 2012). With an assumption of additive effect of exercise, LCHF diet and weight-loss we estimated a difference of 150 U in AUC glucose between groups and a SD of 130, 12 participants were required in each group with a power of 80% with a two-tailed 0.05 significance level. Based on an expected 15% dropout rate, we aimed to recruit 15 participants in each group.

### 2.12 Statistical methods

Descriptive analysis and differences between groups were assessed with t-tests with unequal variances for continuous variables. The variables included age, weight, height, BMI, waist-hip ratio, and blood pressure. Main analyses on outcome variables (glucose tolerance, insulin resistance indicis and android-gynoid fat distribution) were performed with linear mixed models to assess the differences between groups after the intervention. The models included group, time, and group × time interaction set as fixed variables. Measurements were set nested within subject, and time was included as a random slope when improving the model. This was evaluated with a likelihood ratio test. Analyses followed the intention to treat principle; therefore, the last value measured for dropouts was included. We completed pairwise comparisons within (pre-post) and between comparable groups. Differences within groups at post measurements were adjusted for baseline measurements. All pairwise comparisons were also assessed with Bonferroni adjustments due to multiple comparisons. Assumptions were examined with visual inspections of residuals, and model assumptions were considered met. Missing insulin baseline values for three participants were imputed by hot deck imputation. AUC was calculated using the trapezoidal rule (timepoints 0, 20, 40, 60, 90, 120). To calculate Matsuda index, insulinogenic index and the disposition index, 30 min values for glucose and insulin were estimated using the 20 min and 40 min values. Analyses were completed in Stata version 16.1 software (StataCorp. 2019. Stata Statistical Software: Release 16. College Station, TX: StataCorp LCC, Stata, RRID:SCR_012763) with two-sided *p*-values and significance level set to 5%.

## 3 Results

The data reported in this manuscript were collected as part of a larger study. Several the outcomes have been published previously (Valsdottir et al., 2020). Some of the results that were presented in the earlier paper are provided to some degree in the present manuscript to assist with interpretation of the results. For data that have previously been reported the information is stated below tables.

### 3.1 Participant flow

A study flow chart of enrollment and participant flow, as recommended by the Consolidated Standards of Reporting Trials (CONSORT) has been published elsewhere (Valsdottir et al., 2020).

### 3.2 Study participants

Screening took place from October 2013 to January 2014, while the intervention was conducted from January 27 to 7 April 2014. In total, 60 women were eligible for participation; however, three women withdrew during the 3-week run-in with baseline measurements. The intervention thus included 57 Caucasian premenopausal women classified as overweight/obese, aged 33–47 years, who were randomly assigned to one of the four intervention groups. A total of 53 women (93%) completed the intervention, (dropout rate 7%, n = 4). One participant did not adhere to the NORM diet protocol; one withdrew due to a work situation and two gave no reason for withdrawal. Baseline characteristics for participants in each of the intervention groups are shown in Table 1.

**TABLE 1:** Baseline characteristics.

	NORM (n = 15)	LCHF (n = 14)	NORM-EX (n = 14)	LCHF-EX (n = 14)
Variable				
Age (years)	38.6 ± 3.7	40.0 ± 3.0	40.5 ± 3.7	40.8 ± 3.3
Height (cm)	170.7 ± 5.2	169 ± 6.3	170 ± 4.5	164.4 ± 4.4
Weight (kg) pre	89.2 ± 9.2	88.5 ± 7.2	89.4 ± 9.6	87.5 ± 11.2
Weight (kg) post	84.1 ± 9.6	82.0 ± 6.7	83.4 ± 11.4	79.7 ± 11.1
*p-value*	***<0.001***	***<0.001***	***<0.001***	***<0.001***
BMI (kg∙m^−2^) pre	30.7 ± 2.3	30.9 ± 2.6	30.8 ± 2.3	31.6 ± 3.0
BMI (kg∙m^−2^) post	29.0 ± 2.5	28.3 ± 2.0	28.6 ± 2.5	28.8 ± 3.0
*p-value*	***<0.001***	***<0.001***	***<0.001***	***<0.001***
Waist-hip ratio (U) pre	0.88 ± 0.07	0.87 ± 0.07	0.87 ± 0.05	0.89 ± 0.06
Waist-hip ratio (U) post	0.86 ± 0.07	0.84 ± 0.07	0.84 ± 0.04	0.87 ± 0.04
*p-value*	0.044*	***0.002***	***0.002***	0.067
Blood pressure (mmHg)				
Systolic pre	119 ± 15	116 ± 7	114 ± 10	119 ± 14
Systolic post	115 ± 12	113 ± 7	112 ± 10	115 ± 11
*p-value*	0.015*	*0.331*	*0.420*	*0.092*
Diastolic pre	79 ± 10	77 ± 5	76 ± 8	80 ± 9
Diastolic post	77 ± 8	74 ± 5	74 ± 8	75 ± 7
*p-value*	*0.072*	*0.142*	*0.215*	***<0.01***
BMI (kg∙m^−2^)				
<30 pre	8	5	5	5
<30 post	10	8	9	5
30–35 pre	6	7	8	7
30–35 post	5	5	3	7
>35 pre	1	2	1	2
>35 post	0	0	0	0

Data are presented as mean ± standard deviation (SD): Significant p-values marked in bold. BMI, body mass index; NORM, normal diet; LCHF, Low-carbohydrate high-fat diet; NORM-EX, normal diet combined with exercise; LCHF-EX, Low-carbohydrate high-fat diet combined with exercise.

### 3.3 Weight

**FIGURE 2:**
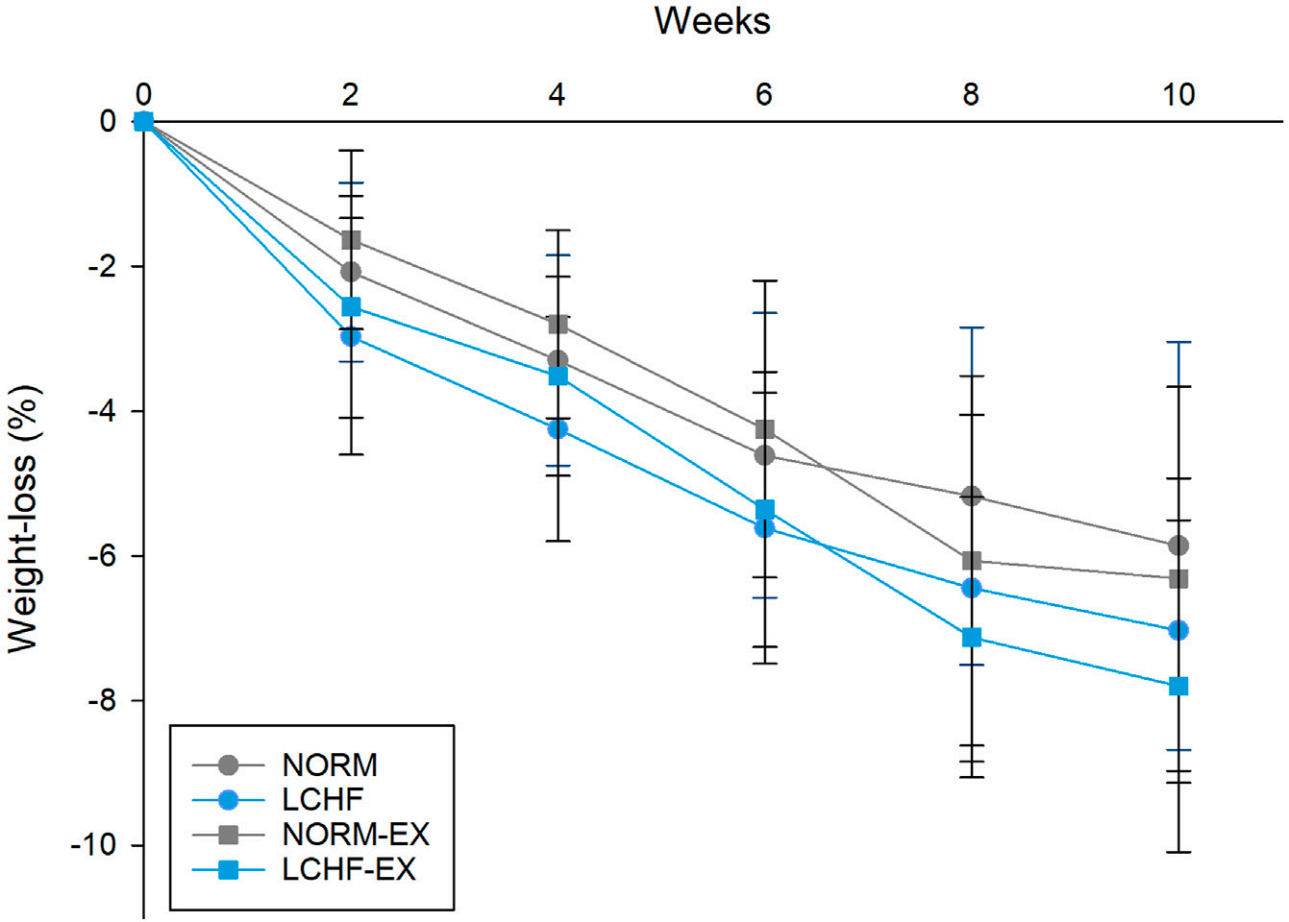
Weight loss during the intervention, measured every 2 weeks. Data are presented as mean ± standard deviation (SD). NORM, Normal diet; LCHF, Low-carbohydrate high-fat diet; NORM-EX, Normal diet combined with exercise; LCHF-EX, Low-carbohydrate high-fat diet combined with exercise.

Detailed data for weight loss have previously been reported (Valsdottir et al., 2020). Briefly, the intervention resulted in similar weight losses in all groups, with no differences between groups. *Within-group* comparison showed that all four groups achieved a weight loss in response to the energy deficit during the intervention. The weight loss was as follows: NORM 5.2 ± 2.3 kg, LCHF lost 6.2 ± 2.1, NORM-EX 5.5 ± 2.2 kg and LCHF-EX 6.7 ± 2.3 kg. The weight loss was 6.7% for all groups pooled (Figure 2).

### 3.4 Cardiorespiratory fitness and exercise compliance

Cardiorespiratory fitness results have previously been reported (Valsdottir et al., 2020). This data is included only to facilitate the interpretation of current results. *Between-group* differences in cardiorespiratory fitness were observed comparing NORM and NORM-EX group (Table 2). The difference was a result of an increase in the NORM-EX group, combined with a decrease in the NORM group. *Within-group* comparison showed a robust increase in the exercise groups in response to the intervention. The NORM-EX group achieved a 10% increase while the LCHF-EX group achieved a 7% increase (Table 2).

**TABLE 2:** Ancillary analysis: Cardiorespiratory fitness at baseline and after the 10-week intervention.

	NORM (n = 15)	LCHF (n = 14)	NORM-EX (n = 14)	LCHF-EX (n = 14)	NORM vs. LCHF	NORM vs. NORM-EX	LCHF vs. LCHF-EX	NORM-EX vs. LCHF-EX
Variable					*p*-value^#^			
V̇O_2peak_ (mL∙min^−1^) pre	2,497 ± 239	2,490 ± 340	2,478 ± 315	2,259 ± 330	0.954	0.847	0.086	0.067
V̇O_2peak_ (mL∙min^−1^) post	2,364 ± 273	2,356 ± 409	2,715 ± 310	2,416 ± 345	0.864	**0.001**	0.311	0.080
*Within-group change (p-value)*	***<0.01***	***<0.001***	***<0.001***	***<0.001***				

Data are presented as mean ± standard deviation (SD): Significant p-values marked in bold.

Cardiorespiratory fitness in mL∙min^−1,^ has previously been published.

#Differences between group at post measurements are adjusted for baseline measurements.

NORM; normal diet, LCHF; Low-carbohydrate high-fat diet, NORM-EX; normal diet combined with exercise, LCHF-EX; Low-carbohydrate high-fat diet combined with exercise.

Training attendance was satisfying in both NORM-EX and LCHF-EX, with 88 ± 7% and 93 ± 7% respectively.

### 3.5 Primary outcome: Glucose tolerance

#### 3.5.1 AUC glucose and insulin

**FIGURE 3:**
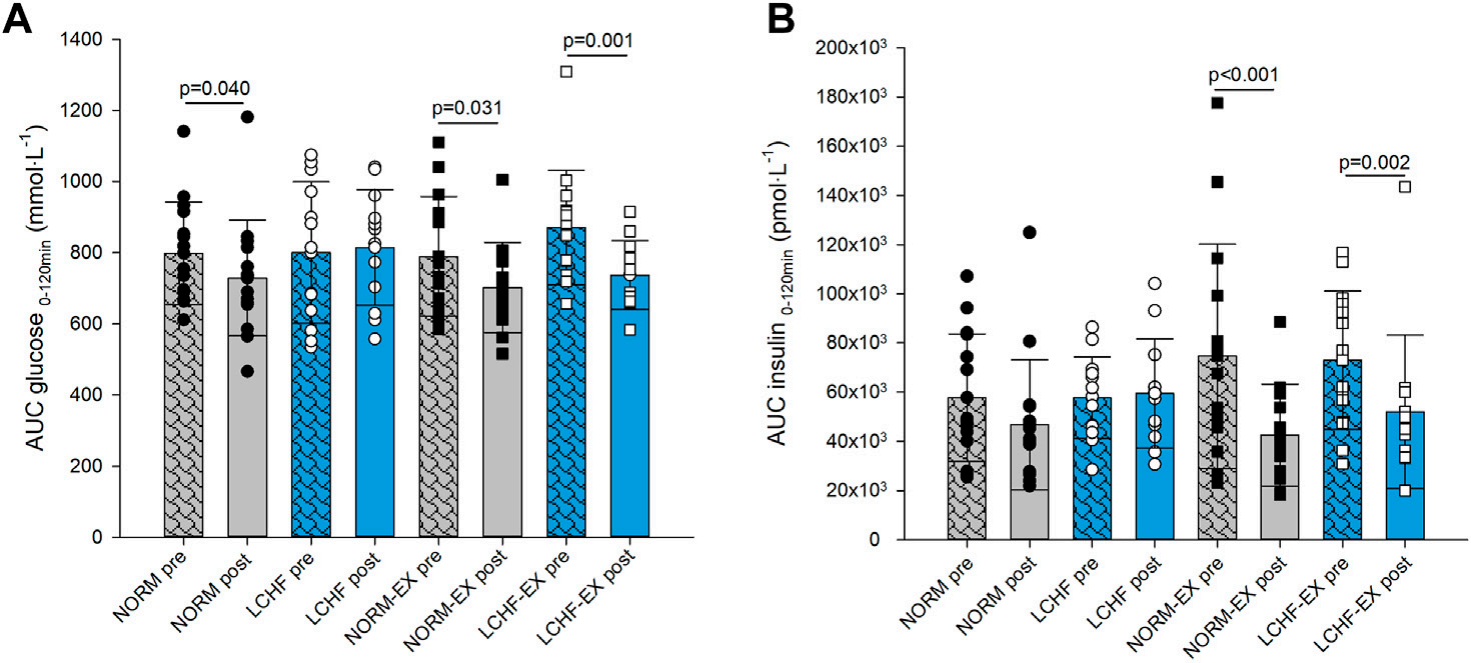
Primary outcome glucose tolerance measured as Area Under Curve (AUC) **(A)** glucose, and **(B)** insulin, during a 120-min oral glucose tolerance test (OGTT) prior to the intervention (pre) and after (post) the 10-week intervention. NORM, Normal diet; LCHF, Low-carbohydrate high-fat diet; NORM-EX, Normal diet combined with exercise; LCHF-EX, Low-carbohydrate high-fat diet combined with exercise.

*Between-group* comparison showed no difference in AUC glucose in response to the 10-week intervention (Figure 3A). *Within-group* comparison showed a 9, 11, and 15% reduction in AUC glucose in NORM, NORM-EX and LCHF-EX respectively. The LCHF group did not achieve a reduction in AUC glucose (*p* = 0.572, Figure 3A). *Between-group* comparison showed no difference in AUC insulin in response to the 10-week intervention (Figure 3B)*. Within-*group comparisons showed a reduction in AUC insulin in the exercise groups NORM-EX (43%) and LCHF-EX (29%), whereas no significant changes were observed in either NORM or LCHF (Figure 3B).

#### 3.5.2 Fasting glucose

*Between-group* comparison showed no difference in fasting glucose after the 10-week intervention (Table 3). *Within-group* comparison showed the LCHF-EX group was the only group to achieve a significant reduction in fasting glucose in response to the intervention, with a decrease of 5%. The other groups did not show any significant response to the intervention (Table 3).

**TABLE 3:** Primary outcome: Fasting values for glucose and insulin, and markers of insulin resistance at baseline and after the 10-week intervention.

	NORM (n = 15)	LCHF (n = 14)	NORM-EX (n = 14)	LCHF-EX (n = 14)	NORM vs. LCHF	NORM vs. NORM-EX	LCHF vs. LCHF-EX	NORM-EX vs. LCHF-EX
Variable					*p*-value^#^			
Fasting values								
Glucose (mmol/L) pre	5.1 ± 0.3	5.1 ± 0.4	4.9 ± 0.2	5.4 ± 0.7	0.738	0.073	0.175	**0.007**
Glucose (mmol/L) post	5.0 ± 0.5	5.0 ± 0.3	4.8 ± 0.3	5.1 ± 0.6	0.828	0.231	0.750	0.261
*Within-group change (p-value)*	*0.453*	*0.304*	*0.652*	***0.007***				
Insulin (pmol/L) pre	74 ± 38	70 ± 26	80 ± 30	88 ± 36	0.737	0.616	0.120	0.504
Insulin (pmol/L) post	69 ± 47	54 ± 31	62 ± 27	73 ± 39	0.283	0.666	0.166	0.458
*Within-group change (p-value)*	*0.554*	*0.131*	*0.082*	*0.111*				
**Insulin resistance**								
HOMA-IR pre	2.35 ± 1.31	2.23 ± 0.98	2.44 ± 0.95	3.00 ± 1.41	0.815	0.845	0.094	0.204
HOMA-IR post	2.24 ± 1.95	1.70 ± 1.04	1.86 ± 0.87	2.38 ± 1.45	0.301	0.506	0.227	0.397
*Within-group change (p-value)*	*0.714*	*0.112*	*0.106*	0.029*				
Matsuda ISI pre	4.91 ± 2.93	4.55 ± 2.21	4.27 ± 2.57	3.60 ± 2.22	0.712	0.543	0.312	0.510
Matsuda ISI post	5.98 ± 2.95	5.31 ± 2.44	6.54 ± 3.25	5.53 ± 3.17	0.462	0.718	0.623	0.568
*Within-group change (p-value)*	0.039*	*0.265*	***<0.001***	***0.001***				
Insulinogenic index pre	1.74 ± 1.04	2.21 ± 1.78	1.79 ± 0.93	1.65 ± 1.23	0.372	0.892	0.327	0.731
Insulinogenic index post	2.72 ± 3.19	2.14 ± 2.12	2.06 ± 1.75	2.99 ± 3.10	0.500	0.440	0.364	0.317
*Within-group change (p-value)*	*0.085*	*0.769*	*0.783*	0.043*				
Disposition index pre	8.27 ± 6.36	11.47 ± 12.05	6.95 ± 4.82	5.31 ± 3.38	0.363	0.532	0.056	0.270
Disposition index post	17.65 ± 23.64	14.94 ± 22.97	12.70 ± 11.48	15.96 ± 21.38	0.668	0.425	0.738	0.407
*Within-group change (p-value)*	0.038*	*0.645*	*0.326*	0.025*				

Data are presented as mean ± standard deviation (SD): Significant p-values marked in bold.

*p*-values marked with * are no longer significant with Bonferroni adjustment.

^#^Differences between group at post measurements are adjusted for baseline measurements.

NORM; normal diet, LCHF; Low-carbohydrate high-fat diet, NORM-EX; normal diet combined with exercise, LCHF-EX; Low-carbohydrate high-fat diet combined with exercise.

#### 3.5.3 Insulin resistance indicis

*Between-group* comparison showed no difference in HOMA-IR after the 10-week intervention (Table 3). *Within-group* comparison showed an improvement in the LCHF-EX group only, with a 20% decrease. No significant changes were observed within the other intervention groups (Table 3).

**FIGURE 4:**
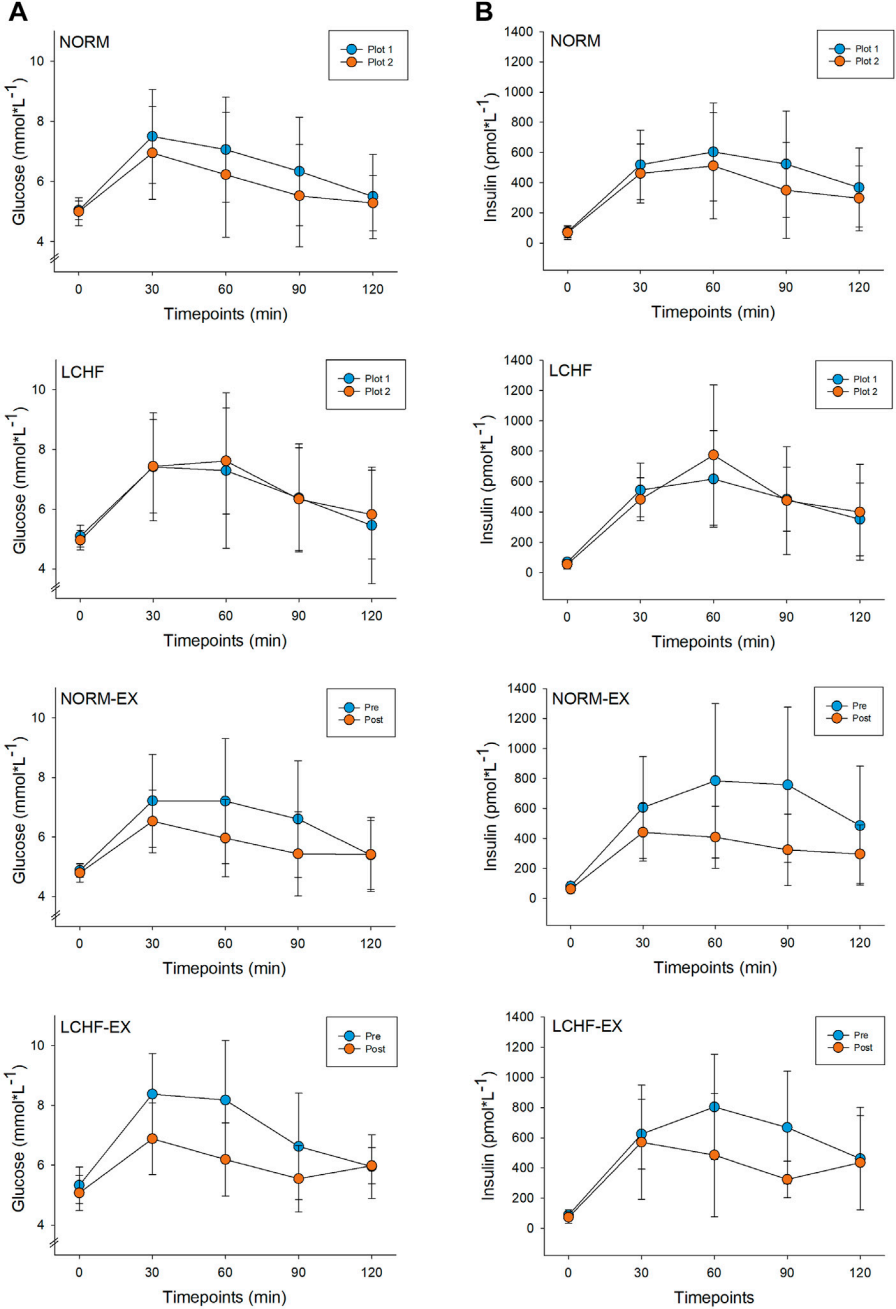
Time course prior to (pre) and after (post) the 10-week intervention for glucose **(A)** and insulin **(B)** during a 120-minute oral glucose tolerance test (OGTT). NORM, Normal diet; LCHF, Low-carbohydrate high-fat diet; NORM-EX, Normal diet combined with exercise; LCHF-EX, Low-carbohydrate high-fat diet combined with exercise.

*Between-group* comparison showed no difference in Matsuda ISI after the 10-week intervention (Table 3; Figures 4A, B). *Within-group* changes in the Matsuda ISI showed an increment of 53% in the NORM-EX, 54% the LCHF-EX, and a 22% increase in the NORM group (Table 3).

*Between-group* comparison showed no differences in neither insulinogenic nor disposition indicis after the intervention (Table 3). The LCHF-EX group was the only group to achieve *within-group* improvement in the insulinogenic index with an 81% increase, whereas both the NORM and the LCHF-EX showed *within-group* improvements in the disposition index, an increase of 113% and 201% respectively.

### 3.6 Secondary outcomes: Distribution of android and gynoid fat, A/G ratio

*Between-group* comparison showed a significantly lower mass of android fat in the LCHF group, when compared with the NORM group after the 10-week intervention (Table 4). *Within-group* comparison showed that all groups achieved a robust improvement in android fat mass in response to the intervention (Table 4), where significant reduction was observed in the NORM group (12%), LCHF group (23%), NORM-EX group (21%) and the LCHF-EX group (20%).

**TABLE 4:** Secondary outcomes: Android fat, gynoid fat, and lean body mass at baseline and after the 10-week intervention.

	NORM (n = 15)	LCHF (n = 14)	NORM-EX (n = 14)	LCHF-EX (n = 14)	NORM vs. LCHF	NORM vs. NORM-EX	LCHF vs. LCHF-EX	NORM-EX vs. LCHF-EX
Variable					*p*-value^#^			
Fat distribution								
Android fat (g) pre	3,664 ± 740	3,287 ± 822	3,446 ± 906	3,765 ± 636	0.163	0.467	0.066	0.274
Android fat (g) post	3,223 ± 727	2,533 ± 535	2,738 ± 856	3,025 ± 703	0.041*	0.294	0.168	0.681
*Within-group change (p-value)*	***<0.001***	***<0.001***	***<0.001***	***<0.001***				
Gynoid fat (g) pre	7,220 ± 1,421	7,184 ± 1,548	6,847 ± 1,213	6,930 ± 1,252	0.945	0.443	0.600	0.859
Gynoid fat (g) post	6,736 ± 1,413	6,308 ± 1,172	6,018 ± 1,458	5,977 ± 1,415	0.287	0.145	0.587	0.902
*Within-group change (p-value)*	***<0.001***	***<0.001***	***<0.001***	***<0.001***				
A/G ratio pre	1.06 ± 0.08	0.99 ± 0.11	1.05 ± 0.11	1.11 ± 0.07	**0.042**	0.785	**0.001**	0.089
A/G ratio post	1.04 ± 0.09	0.94 ± 0.12	1.04 ± 0.14	1.09 ± 0.09	0.011*	0.702	**<0.001**	0.527
*Within-group change (p-value)*	0.030*	***<0.001***	*0.968*	0.039*				
Lean body mass (kg) pre	47.9 ± 4.4	48.1 ± 3.9	49.0 ± 4.5	45.9 ± 5.3	0.869	0.471	0.192	0.082
Lean body mass (kg) post	46.1 ± 4.3	47.2 ± 4.3	48.2 ± 5.0	44.2 ± 4.8	0.546	0.234	0.201	0.103
*Within-group change (p-value)*	***<0.001***	***<0.001***	***<0.001***	***0.001***				

Data are presented as mean ± standard deviation (SD): Significant p-values marked in bold.

*p*-values marked with * are no longer significant with Bonferroni adjustment.

^#^Differences between group at post measurements are adjusted for baseline measurements.

LBM (Lean body mass) (kg) has previously been published. Results are presented to help with interpretation of other results.

NORM, normal diet; LCHF, Low-carbohydrate high-fat diet; NORM-EX, normal diet combined with exercise; LCHF-EX, Low-carbohydrate high-fat diet combined with exercise.

A/G ratio, android/gynoid ratio.

*Between-group* comparison showed no difference in gynoid fat mass after the 10-week intervention (Table 4). *Within-group* comparison showed that all groups achieved a reduction in gynoid fat mass in response to the intervention (Table 4). Significant reduction was observed in the NORM group (7%), LCHF group (12%), NORM-EX group (12%) and LCHF-EX group (14%).

*Between-group* comparison showed a significantly lower A/G ratio in the LCHF group, when compared with the NORM, and when comparing the LCHF and LCHF-EX groups (Table 4). *Within-group* reduction was observed in the NORM group (2%), the LCHF group (5%), and the LCHF-EX group (2%), with no changes in the NORM-EX group (Table 4).

### 3.7 Harms or unintended effects

No unintended or serious effects were reported. Well-known minor and non-serious side effects of the LCHF diet were reported during the first 2 weeks. Those were dizziness (n = 19), headache (n = 12) and lack of power during exercise sessions (n = 8).

## 4 Discussion

### 4.1 Primary outcome: Glucose tolerance

The main finding in our study was that weight loss achieved with combined LCHF diet and exercise, caused no superior improvement in glucose tolerance after a 10-week intervention. Indeed, the intervention resulted in no differences in glucose tolerance when comparing the intervention groups. Nevertheless, a significant *within-group* improvement in AUC glucose was observed in all intervention groups, except the LCHF group (*p* = 0.572).

#### 4.1.1 AUC glucose

The results indicate that improvements in glucose tolerance, measured as AUC, are not attributable to either specific diet or exercise. Rather, the improvements seem to be a combined effect of exercise and diet resulting in weight loss. Our results are in line with previous studies, showing no improvements in AUC glucose when comparing a calorie restricted diet and a calorie restricted diet plus exercise (Schenk et al., 2009; Keshel and Coker, 2015; Weiss et al., 2015). Prior to the study, we speculated an additive effect of weight loss, exercise and LCHF diet on glucose tolerance. This hypothesis was based on previous findings that separately showed positive effect on glucose tolerance by exercise (Jenkins and Hagberg, 2011; Bird and Hawley, 2016; Malin et al., 2016; De Strijcker et al., 2018), calorie deficit resulting in weight loss (Pate et al., 1995; Norris et al., 2005; Schenk et al., 2009) and a LCHF diet (Noakes and Windt, 2017). Therefore, we anticipated significant improvements in the exercise groups compared to diet-only groups, with superior effects on glucose tolerance in LCHF-EX. However, our study did not reveal differences among the groups, presumably as a result of smaller improvements in AUC glucose than hypothesized.

Within-group comparisons showed improved AUC glucose in both exercise groups, in addition to the NORM group. Our lab has previously shown a 12% improvement in AUC glucose in young females with normal weight ingesting normal diet, after a bout of exercise (Valsdottir et al., 2019). Results from the present study show that the three groups with a significant reduction in AUC glucose had post levels close to baseline levels in females with normal weight. The positive effect of exercise on glucose tolerance is short lived and transient and must be maintained with repeated bouts of exercise, with no longer than 48–72 h between sessions The present results indicate that improvements in glucose tolerance are relatively long-lived and detectable after 36 h post exercise in this population. Nevertheless, the effects of exercise were possibly starting to fade in the exercise groups. This indicates that a bout of exercise must be repeated regularly to maintain improvement in glucose tolerance, and possibly achieve chronic improvement. It is noteworthy that the NORM group exhibited AUC values within the normal range after the intervention (Valsdottir et al., 2019), suggesting that the E% from carbohydrates may be essential to maintain metabolic flexibility and the ability to handle glucose loads, as no improvement was observed in LCHF despite a 7.1% weight loss. The lack of improvement in glucose tolerance in the LCHF group, contrary to improvements in the NORM group, can be explained by decreases in rates of carbohydrate oxidation due to adaptation to the LCHF diet. This is supported by Burke et al. (2000) who observed persistent high fat oxidation despite restoration of carbohydrate availability after a LCHF diet. Notably, Kirk et al. (2009) concluded that 11 weeks of an LCHF diet increased both hepatic and skeletal muscle insulin sensitivity, measured with euglycemic-hyperinsulinemic clamp. These discrepancies can be attributed to the larger fat mass in the participants in that study, and a larger calorie deficit (−1,000 kcal/day) prescribed, as improvements in glucose tolerance are easier to achieve when BMI is higher (Bird and Hawley, 2016).

The improvements in AUC glucose in NORM and NORM-EX were 9% and 11%, respectively; still quite far from the 15% improvement in LCHF-EX. The robust improvement observed in LCHF-EX may relate to the positive effect of exercise on glucose uptake in skeletal muscle (Rose and Richter, 2005), as the comparable LCHF did not show any improvement in glucose tolerance. The reduction in AUC in LCHF-EX was expected in response to the exercise, and in accordance with previous observations in overweight males (Jelstad et al., 2019). The results from that project formed the basis for the sample-size calculations in this study. However, improvements in glucose tolerance may be gender-specific, as Metcalfe et al. (2012) did not see any improvement in AUC glucose in females after 18 high-intensity interval sessions across 6 weeks. The various results in AUC glucose after lifestyle interventions can be related to several factors, including initial body weight, total weight loss, gender, age, exercise intensity and timing of glucose tolerance testing after the last bout of exercise.

#### 4.1.2 AUC insulin

AUC insulin showed a similar pattern to AUC glucose in this study, with no differences among the groups. However, a robust *within-group* reduction was observed in both exercise groups, with no improvement in the diet-only groups. As exercise stimulates non-insulin-dependent glucose uptake (Kjobsted et al., 2018), it is legitimate to attribute the reduction in AUC insulin to the regular exercise during the 10-week intervention. Considering the lack of difference *between-group*s, we cannot conclude that inclusion of exercise in weight-loss programs will improve AUC insulin in this population. Previous studies with regular exercise for participants with overweight and obesity have shown improvements in AUC insulin (Jelstad et al., 2019; Bergman and Goodpaster, 2020). However, the positive effect of exercise on glucose disposal is essential, as the reduction of pancreatic secretion of insulin may be an important factor in preventing T2DM later on in life. High production of insulin over time has been linked to reduced function of ß-cells and pancreatic failure (Abdul-Ghani and DeFronzo, 2009).

#### 4.1.3 Fasting glucose

This 10-week intervention provided divergent results in terms of fasting glucose among the intervention groups, although no *between-group* differences were observed. LCHF diets with weight loss <5% have shown improvements in fasting glucose in females with normal weight (Valsdottir et al., 2019) and in individuals who are overweight/obese (Hall and Chung, 2018). Improvements in fasting glucose have been seen in response to both a bout of exercise and prolonged exercise program, with and without weight loss (Keshel and Coker, 2015). Nevertheless, our study showed no effect of exercise on fasting glucose, as no difference was seen between the LCHF and LCHF-EX nor NORM and NORM-EX groups.

Improved fasting glucose is one of the most noticeable responses to weight reduction (Clamp et al., 2017; Gilbertson et al., 2019). In weight-loss studies of subjects with prediabetes, low-carbohydrate diets have been superior to low-fat diets in lowering fasting glucose (Kirkpatrick et al., 2020). However, after this period the differences faded, allegedly a consequence of the gradual increase in carbohydrate intake in most LCHF diets, after the induction ketosis phase (Atkins, 2002; van Wyk et al., 2016; Kirkpatrick et al., 2020). When the carbohydrate intake reaches 150–200 g (or 25 E%), the diet becomes a moderate-carbohydrate diet (Wylie-Rosett et al., 2013; Kirkpatrick et al., 2020), priming the cells and rerouting the metabolism back to carbohydrate oxidation (Burke et al., 2000). Others have shown an additive effect of diet and exercise for reducing fasting glucose (Weiss et al., 2015; Weiss et al., 2016). However, this was not the case in our study, despite our participants staying well below 150 g of carbohydrates throughout the intervention. Several factor can explain lack of differences between groups, such as normoglycemic participants at baseline (Serra et al., 2017), persistent high BMI (Zhu et al., 2019) and high percentage of bodyfat (Wiklund et al., 2008). In addition, despite a low average intake of carbohydrates during the intervention, the average amount during the last week was 25% in LCHF and 31% in LCHF-EX. These amounts are equal to, and above the limits for LCHF diets and may possibly change the positive effects previously seen on fasting glucose during LCHF diets.

Regardless of the absence of *between-group* differences, we observed *within-group* improvements in fasting glucose in the LCHF-EX group. Others have shown that both LCHF diets and exercise had positive effects and reduced fasting glucose (Kirkpatrick et al., 2020). While LCHF-EX was the only group to achieve improvements in the current study, it must be emphasized that this group also showed the highest level of fasting glucose prior to the intervention and reached baseline levels comparable the other groups, after the intervention. This is in line with previous LCHF studies in females with T2DM and overweight/obesity (carbohydrates <30 g) (Hallberg et al., 2018) and healthy females with overweight/obesity (8 E% from carbohydrates) (Michalczyk et al., 2020). On the contrary, Shai et al. (2008) reported no improvements in fasting glucose following a LCHF diet with 20 g of carbohydrates per day during an induction phase, followed by a gradual increase up to 120 g per day (40 E%) throughout the intervention.

The denominator for large improvements in fasting glucose seems to be high glucose at baseline, giving room for more pronounced reduction in response to an intervention and possibly explaining the lack of improvement in the LCHF diet group *versus* the comparable exercise group (LCHF-EX), without *between-group* differences. Our protocol included testing towards the lower end for positive effect of exercise, and it is therefore plausible that the only group that achieved positive effect was the one with the most unfavorable baseline levels.

All groups were within the normal reference range prior to the intervention, and improvements in normal values are not decisive for primary T2DM prevention.

#### 4.1.4 Fasting insulin

Baseline measurements showed normal insulin levels in all groups, as normoglycemic subjects have a mean value of 72 (48–102) pmol/L (Festa et al., 2008). The intervention did not result in differences between groups, and no improvements were seen within groups. This is in line with Gilbertson et al. (2019), where participants ingested normal diets and protein-rich meal replacements. However, Weiss et al. (2016) observed between-group reduction in insulin after a 6%–8% weight loss in three intervention groups (calorie restriction, exercise, and calorie restriction plus exercise). Fasting insulin levels however, are associated with large individual variations without individuals being insulin resistant or having reduced glucose tolerance (Festa et al., 2008).

#### 4.1.5 Insulin resistance indicis

LCHF-EX was the only group to attain a significant improvement in HOMA-IR despite equal weight loss in all groups. Nevertheless, LCHF-EX was the only group to exhibit HOMA-IR *above* cut-off values of 2.29 (Radikova et al., 2006) after the intervention, reflecting a persisting hepatic insulin resistance in this group. The low and non-significant improvements in glucose and insulin in the other groups are reflected in the HOMA-IR, and similar lack of improvement has previously been observed by Gilbertson et al. (2019) in a short-term study with diet-only, and diet and exercise protocols.

Despite the lack of significant improvements within NORM, LCHF and NORM-EX, these groups achieved a reduction in HOMA-IR, with post values *below* the cut-off point for hepatic insulin resistance (Radikova et al., 2006). This demonstrates a beneficial impact on insulin sensitivity and cardiometabolic health (Hallberg et al., 2018), which is an important factor in primary prevention (Ebbeling et al., 2022).

The Matsuda ISI gives a dynamic picture of the glucose disposal during an OGTT and sheds light on the body’s ability to handle a glucose load, rather than insulin sensitivity *per se*. Figure 4 shows a) glucose and b) insulin time course for all groups, pre and post intervention. The Matsuda ISI is used to estimate peripheral (skeletal muscle) insulin sensitivity (Matsuda and DeFronzo, 1999). The intervention did not result in *between-group* differences in the Matsuda ISI. However, w*ithin-group* improvement was observed in the exercise groups, in addition to the NORM group. In addition, all groups to reached values higher than the cut-off level of 5, which is regarded as appropriate to maintain a healthy insulin sensitivity. The large within-group increases of 53% (NORM-EX, *p* = 0.001) and 54% (LCHF-EX, *p* = 0.001) in the exercise groups bear great resemblance to the findings of Weiss et al. (2015). Our results show that weight-loss with or without exercise increases peripheral insulin sensitivity. Yet, due to the lack of between-group differences we cannot state that exercise has superior effect than diet only. Noteworthy, our study was powered for AUC glucose as primary outcome so considering the larger increase in Matsuda ISI the exercise groups, it is plausible that the inclusion of exercise in lifestyle interventions should be preferred to achieve weight loss and improve insulin sensitivity.

Prior to the intervention, all groups were above the insulinogenic index threshold of ≥0.4, and the 10-week intervention did not result in *between-group* differences The LCHF-EX group was the only group to show a *within-group* improvement in response to the intervention (81%). The insulinogenic index is used as an index for early phase insulin secretion and is a reasonable surrogate for acute insulin response (AIR) (Aono et al., 2018). However, improvements above the normal baseline levels can be difficult to reach.

No *between-group* differences were in the disposition index after the intervention, and only two of the groups showed an improvement in response to the treatment; NORM (113%) and the LCHF-EX (201%). The disposition index can be used to assess β-cell function during an OGTT and identifies β-cells deficiency and the inability to compensate for insulin resistance. A low disposition index is an early marker of faulty β-cells and predicts a development to T2DM, beyond fasting glucose levels (Abdul-Ghani et al., 2007; Utzschneider et al., 2009). Previous studies have shown that the disposition index and first-phase insulin are not affected when adjusted for visceral adiposity and BMI (Burns et al., 2011), suggesting that the participants’ adiposity after the intervention may play a significant role in the absence of improvement in the insulinogenic and disposition index.

### 4.2 Secondary outcome: Android and gynoid fat, A/G ratio

After the intervention, between-group comparisons showed larger reductions in android fat mass in LCHF compared to NORM, indicating a positive effect of the LCHF diet. However, this did not affect the glucose tolerance positively. Differences in android fat mass were observed between NORM and LCHF, but not between NORM-EX and LCHF-EX. Hence, we cannot attribute any positive effects of the LCHF diet on android fat, or on the primary outcome glucose tolerance. This is supported by previous research where high-carbohydrate and high-fat diets did not differentially influence android visceral fat area (Veum et al., 2017). Android obesity in females has been related to reduced insulin sensitivity (Wiklund et al., 2008), and reduction in android fat levels should therefore lead to improved glucose tolerance. Nevertheless, even with substantial improvement in android fat in LCHF, no improvement was observed in glucose tolerance in this group. Previous research has shown that android and gynoid fat have opposite associations with CVD and other metabolic risk factors (Lumish et al., 2020), and higher distribution of gynoid fat is thought to serve as a protection against CVD (Wiklund et al., 2008). It should be noted that gynoid fat as compartment often reflects a linear relationship with total fatness and increased CVD risk (Fox et al., 2007; Wiklund et al., 2008). Indeed, studies of females with normal weight have shown that joint occurrence of elevated android and gynoid fat percentage is associated with higher odds for elevated glucose than high android fat alone (Okosun et al., 2015). The present intervention resulted in a substantial reduction in both android and gynoid fat in all groups. However, the persisting high percentage of body fat, likely prevented a significant improvement in glucose tolerance, as gradients of adiposity have been shown to increase the numbers of CVD risk factors (Okosun et al., 2015; Lüscher, 2019; Sun J. et al., 2022), specifically in women, where the strongest associations between insulin resistance and A/G ratio are observed (Bantle et al., 2019). At the same time, the reduction in A/G ratio in response to the intervention showed a favorable shift towards a healthier ratio, yet the magnitude of the reduction did not reach the average healthy female ratio of 0.79.

### 4.3 Ancillary analysis

#### 4.3.1 Weight

Data for weight have been published elsewhere (Valsdottir et al., 2020). The weight loss was controlled throughout the intervention, and a target of 5% was achieved in all groups, where multiple pooling of exercise and diet groups showed no differences in weight-loss. However, large interindividual differences were observed, and 16 participants (29%) did not reach the 5% target despite self-reported adequate calorie deficit. Further on, eight of the participants (14%) did not reach 4%, whereas only three participants (5%) did not reach a 3% weight-loss. This can be explained by several factors, such as overestimating PAL and calorie requirement at baseline and a greater energy intake than assessed by diet records. Previous studies have observed some underreporting, with a greater bias in females and individual who are obese and weight-conscious (Schoeller, 1995; Millen et al., 2009; Stubbs et al., 2014). However, one should not criticize participants for underreporting when “calories-in calories-out” calculations do not give results as expected. Recent studies have unveiled a possible link between the gut microbiome and weight gain (Aoun et al., 2020), suggesting microbes in the intestine can impact the absorption, breakdown and storage of nutrients. Others have suggested that energy deficit results in adaptive reduction in thermogenesis and resistance to losing weight (Muller et al., 2016). Although the main factor conceded during weight-loss, is adherence to the prescribed energy deficit, the genetic component will influence the ability to respond. The development of overweight and obesity has a strong genetic component which also can cause resistance to lose weight (Lamiquiz-Moneo et al., 2019). Further on, Bouchard et al. (1994) demonstrated individual differences in reduction of both subcutaneous and visceral fat mass in response to the same amount of exercise, indicating genetic differences. All aforementioned factors are plausible, but outside the scope of this manuscript.

#### 4.3.2 Cardiorespiratory fitness

The increase in cardiorespiratory fitness in the exercise groups is in accordance with the exercise implemented in the exercise sessions, as previous studies have shown positive effects of varying interval exercises on cardiovascular fitness in participants with overweight/obesity (Kuehnbaum et al., 2014; Sawyer et al., 2016; Andrade-Mayorga et al., 2021). In the present study we observed a reduction in V̇O_2peak_ mL min^−1^ in the non-exercising groups. A reduction in cardiorespiratory fitness after weight-loss without exercise has been observed by others (Goran et al., 2000). The reduction appears to develop due to reduced body mass that results in lesser cardiorespiratory demand during daily activities. Low cardiorespiratory fitness is associated with a two-fold higher risk of premature mortality in men, independent of BMI/fatness (Tarp et al., 2021). A similar pattern is evident in women, although not detectable in conservative models. Despite weaker association in women, the potential modifying role of cardiorespiratory fitness on obesity mortality supports the inclusion of sufficient physical activity in lifestyle interventions.

### 4.4 Strength and limitations

The strengths of this study are the inclusion of females only, and the high compliance with the exercise program. Another strength is the tight supervision of both diet and exercise.

Some limitations must also be acknowledged. In our power calculation we anticipated an improvement in AUC of 150 ± 130 (mean ± SD). However, the greatest improvement in AUC was 134 U in LCHF-EX. Due to this modest improvement, no between-group differences were detected. Hence, it can be argued that this study was underpowered to detect such modest improvements, potentially resulting in type II error. Another limitation is that even though the average weight loss achieved for all groups was acceptable, we assume that all participants would have had to reach the target of 5% weight loss to induce improvements in glucose tolerance. Weiss et al. (2015) had previously an extended intervention period until all participants achieved the targeted weight loss. Unfortunately, this was not possible with the present design, as increased exercise sessions in the exercise groups would induce larger effects on parameters linked to weight-loss and cardiorespiratory fitness. Another study limitation is that the exercise groups got additional interactions compared to the diet-only groups, as these participants both mingled and met with the staff and researchers three times weekly during exercise sessions. This study did not control for the greater amount of personal contact time received by the exercise groups relative to the diet-only groups. Moreover, we did not control the timing of testing relative to menstrual phase, which may increase variability in glucose tolerance (MacGregor et al., 2021). In view of the positive effect of increased physical activity during the intervention, the lack of monitoring daily physical activity of participants must be considered as a limitation in this study.

## 5 Conclusion

Matched weight loss during a 10-week program with diet only, or with a combination of exercise and diet, resulted in improvements exclusively in the exercise groups, in terms of cardiorespiratory fitness and AUC insulin. Collectively, these results emphasize the positive effects and importance of exercise during a weight-loss program.

As the current study was designed to compare the effectiveness of the intervention groups, the main conclusion for *between-group* comparisons showed no superior effect for any of the intervention groups with regard to the primary outcome glucose tolerance (AUC glucose). Thus, the beneficial effects of exercise in women with overweight/obesity extend beyond the adaptive response to a single outcome variable such as fat distribution, glucose tolerance or weight reduction.

## Data Availability

The raw data supporting the conclusions of this article will be made available by the authors, without undue reservation.
